# The tumor burden score may be a discriminator in microwave ablation versus liver resection for hepatocellular carcinoma within the Milan criteria: a propensity score matching and inverse probability of treatment weighting study

**DOI:** 10.3389/fonc.2024.1330851

**Published:** 2024-02-16

**Authors:** Zeyuan Wei, Kailing Xie, Feng Xu, Chaoliu Dai

**Affiliations:** ^1^ Department of Hepatobiliary and Splenic Surgery, Shengjing Hospital of China Medical University, Shenyang, China; ^2^ Department of General Surgery, Shengjing Hospital of China Medical University, Shenyang, China

**Keywords:** liver resection, microwave ablation, hepatocellular carcinoma, tumor burden score, prognosis

## Abstract

**Purpose:**

This study aims to compare the prognostic outcome of resection (RES) and microwave ablation (MWA) in different tumor burden score (TBS) cohorts.

**Patients and Methods:**

We retrospectively analyzed 479 patients with primary hepatocellular carcinoma (HCC) who underwent RES (n = 329) or MWA (n = 150) with curative intent at our institution. We assessed their overall survival (OS) and progression-free survival (PFS) using the Kaplan–Meier curve. Propensity score matching (PSM) and inverse probability of treatment weighting (IPTW) were performed to minimize selection and confounding biases. Multivariate Cox regression was used to define the association between surgical modalities and outcomes.

**Results:**

Following PSM, in the TBS ≤3 cohort, the cumulative 1-, 3-, 5- year OS in the RES and MWA groups were 92.5% vs. 98.8%, 82.7% vs. 90.0%, and 82.7% vs. 83.2% (P = 0.366), respectively. The corresponding PFS rates in the RES and MWA groups were 82.7% vs. 88.0%, 63.6% vs. 68.3% and 55.2% vs. 56.3, respectively (P = 0.218). In the TBS >3 cohort, the cumulative 1-, 3-, 5- year OS between the RES and MWA groups were 92.5% vs. 95.0%, 82.8% vs. 73.2% and 76.3% vs. 55.1%, (P = 0.034), respectively. The corresponding PFS rates in the RES and MWA groups were 78.0% vs. 67.5%, 63.6% vs. 37.5% and 55.2% vs. 37.1%, respectively (P = 0.044). The IPTW analysis showed similar results as shown in PSM analysis. The multivariate Cox regression indicated that the type of surgical modality was not associated with a poorer prognostic outcome in the TBS ≤3 cohort, unlike in the TBS >3 cohort.

**Conclusion:**

TBS, as a discriminator, might help guide treatment decision-making for HCC within the Milan criteria.

## Introduction

Hepatocellular carcinoma (HCC) remains a major clinical challenge considering its relatively high prevalence, rapid progression, and dismal prognosis (1). With improved screening strategies and elevated public awareness, HCC can be detected early and properly managed. Currently, liver resection, liver transplantation, and ablation therapy, such as radiofrequency ablation and microwave ablation (MWA), are the main curative treatments for early-stage HCC (2). Resection was regarded as the standard first-line treatment for those with small primary liver cancer, but some patients are not candidates due to impaired general health status, hepatic insufficiency, and insufficient residual liver volume (3). MWA is an ablation modality that destroys cancer cells using heat from microwave energy. With the rapid advancements and breakthroughs, MWA has become indispensable for managing small HCCs due to its safety, minimal invasiveness, lower expense, and rapid recovery time (4).

Nevertheless, the best management approach for small primary liver cancers eligible for microwave coagulation and liver resection remains controversial. Several studies have compared the prognosis among patients with HCC treated with MWA and surgical resection. Results revealed that MWA was comparable with liver resection in terms of the 1-, 3-, and 5-year OS, and recurrence-free survival (5, 6). However, a systematic review and meta-analysis indicated that the local ablation group demonstrated a significantly higher incidence of local recurrence than the liver resection group (7).

Of note, tumor burden largely indicates the extent of the tumor in HCC and is included in the Barcelona Clinic Liver Cancer (BCLC) and other staging systems (8). Previous studies have shown that differences in tumor characteristics, such as size, and number, affect the outcomes, and prognosis of resection and ablation therapy (9–11). The tumor burden score (TBS), calculated by combining the maximum tumor size and number of lesions, was proposed to predict survival in colorectal liver metastasis (12). TBS was later applied to stratify the prognosis for patients with HCC undergoing resection, local ablation, and transarterial chemoembolization (13–16) and demonstrated a better discriminative ability than the Milan criteria (17). A recent study by Ho et al. showed that TBS was a promising marker to discriminate long-term outcomes in patients with HCC within the Milan criteria undergoing local ablation or transarterial chemoembolization (18). Building on these previous findings, this study aimed to investigate the possible role of preoperative TBS in discriminating the therapeutic outcomes of MWA and liver resection in HCC within the Milan criteria.

## Materials and methods

### Patients

We retrospectively collected the data of patients with primary HCC who underwent resection or MWA with curative intent from 2012 to 2019 at the Department of General Surgery, Shengjing Hospital of China Medical University. All patients were diagnosed with HCC preoperatively via contrast ultrasound (US) and enhanced multidetector computed tomography (CT) and magnetic resonance imaging (MRI) accompanied by the alpha-fetoprotein (AFP) test. The inclusion criteria were as follows: (a) within the Milan criteria, (i) single tumor lesion ≤5 cm in diameter or multiple tumor nodules (two to three) and a maximum diameter ≤3 cm, (ii) no vessel or bile duct invasion, and (iii) no lymph node or extrahepatic metastasis; (b) R0 resection; and (c) complete ablation (defined as an ablation zone with a margin [≥5 mm] covering the original tumor size and no HCC features in the imaging test postoperatively). The exclusion criteria were (a) patients with cardiovascular or immune system disease; (b) Child–Pugh score C; (c) repeated carcinoma; (d) ablation combined with resection; (e) loss to follow-up; and (f) lack of laboratory data.

Demographic characteristics and clinical features included sex; age; body mass index (BMI); AFP level; neutrophil-to-lymphocyte ratio (NLR); hepatopathy; platelet (PLT) count; albumin (ALB), total bilirubin (TBIL), alanine aminotransferase (ALT), and aspartate aminotransferase (AST) levels; prothrombin time (PT); Child–Pugh score; maximum tumor size; tumor number; cirrhosis; hypertension; and hypersplenism. The study was performed in accordance with the ethical guidelines of the Declaration of Helsinki and approved by the Ethics Committee of Shengjing Hospital of China Medical University (2023PS760K). Written informed consent was obtained from all the patients or their representatives. To protect patient privacy, we de-identified all data that can be used to identify patient personal information, such as name, hospital ID, and telephone number.

### Treatment and follow-up

For the resection group, a team of dedicated liver surgeons performed liver resection. The type and extent of resection were based on the extent of the tumor and hepatic functional reserve. Anatomical resection was the primary option, whereas non-anatomical resection was the secondary option if anatomical resection was not feasible. Intraoperative ultrasonography was used to achieve a resection margin of at least 1 cm. Liver parenchymal dissection was performed using bipolar coagulation forceps (SY-VIIC(Q)-6, Zhejiang, China), harmonic scalpel (HARMONIC SYNERGY® Blades, Ethicon Inc., Cornelia, GA), or the clamp-crushing method with an intermittent Pringle maneuver that was routinely performed within 15 min of ischemia, followed by 5 min of reperfusion.

For the MWA group, MWA was performed using a cooled-shaft system (ECO-100AI10, ECO Microwave System Co, Nanjing, China) with a maximum power of 80 W at 2450 MHz at our institution. The system was equipped with a real-time temperature monitor and cooling circulation technology. The operation was performed by a hepatobiliary surgeon (5–10 years of experience in MWA). All patients underwent US to locate tumor lesions and determine the best treatment strategy. For tumors with a diameter within 2 cm, the antenna was placed at the center of the tumor. For the tumors with a diameter of 2 to 3 cm, the antenna was placed on both sides of the tumor. For the tumors with a diameter exceeding 3 cm, multiple overlapping ablations were performed by repositioning the antenna. The antenna was placed sequentially on different areas of tumors according to the tumor size and shape. The surgeons tried to achieve complete tumor ablation with a margin exceeding 1 cm.

All patients were followed up with CT, MRI, and US 2 months after surgery, and AFP levels were monitored, with follow-up every 3 months thereafter. Treatment options for relapsed patients were determined on the basis of tumor number, size, and location and the patient’s liver function status. Overall survival (OS) was the survival time from the end of the initial surgery to death or the last follow-up. Progression**-**free survival (PFS) was the survival time from the end of the initial surgery to the first discovery of tumor recurrence or the last follow-up.

### TBS

According to previous reports, TBS is defined as the distance from the origin of a Cartesian plane and comprises two variables: maximum tumor size (x-axis) and number of tumors (y-axis); thus, TBS^2^ = (maximum tumor diameter)^2^ + (number of tumors)^2^. The cutoff value of TBS was 3.0.

### Propensity score matching and inverse probability of treatment weighting

PSM and IPTW were used to minimize selection and confounding biases. The PS was calculated using logistic regression with the following clinical features: sex; age; BMI; NLR; the presence of viral hepatitis; AFP, ALT, and AST levels; PT; Child–Pugh score; the presence of cirrhosis; the presence of hypersplenism.

In the TBS ≤3 cohort, PSM was performed using a ratio of 1:1 via the nearest neighbor matching algorithm with an optimal caliper of 0.2 for MWA versus resection. In the TBS >3 cohort, PSM was performed using a ratio of 1:4 via the nearest neighbor matching algorithm with an optimal caliper of 0.2 for MWA versus resection.

For stabilized IPTW, the weighting coefficient of patients in the MWA and resection groups was PT/PS and (1 − PT)/(1 − PS)(PT = patients in the MWA group/all patients), respectively.

### Other statistical analyses

For continuous variables, if they conformed to the normal distribution, the data were presented as the mean ± standard deviation; otherwise, they were presented as the median (quartile 25%, 75%). If the continuous data satisfied normality, a comparison between the two groups was analyzed with the t-test; otherwise, a nonparametric test was used. Fisher’s exact or the chi-squared test was used to compare categorical variables. PFS and OS were assessed using the Kaplan–Meier method with a log-rank test. The association of clinicopathologic variables with PFS and OS was assessed with univariate Cox proportional hazards regression. The multivariate Cox proportional hazards regression model was created with statistically significant (P< 0.05) and clinically relevant (P < 0.1) variables. The “survival” and “survminer” packages were used for survival analyses. The “ggplot2” package was used for plotting. The “RISCA” package was used for IPTW. R version 4.1.2 (R Foundation for Statistical Computing, Vienna, Austria) and SPSS version 26.0 (SPSS, Chicago, IL) were used for statistical analyses. A two-tailed P-value of <0.05 was considered statistically significant.

## Results

### Patients

**Figure 1:**
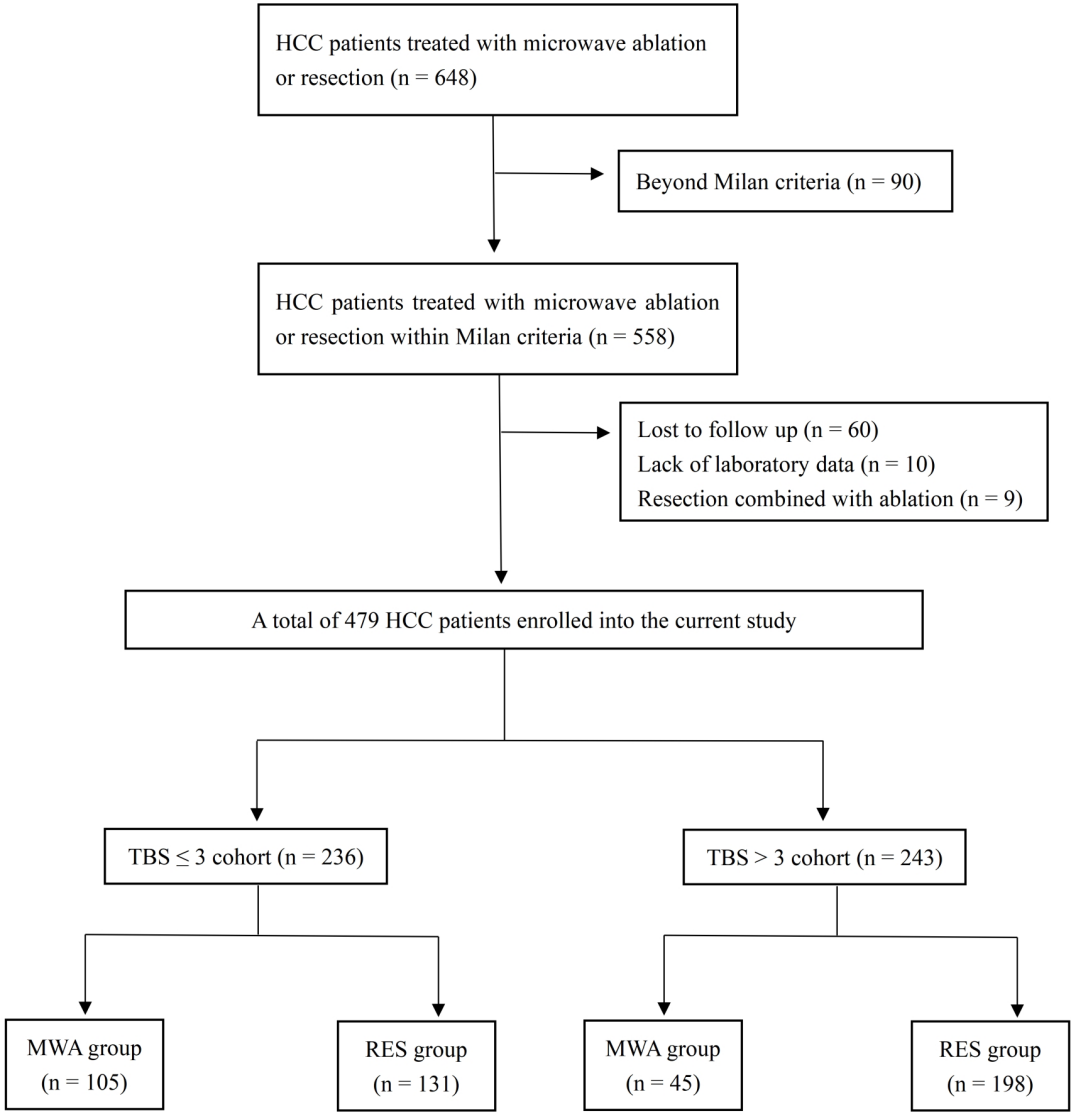
Flowchart of patient selection.

The current study enrolled 479 patients (men and women, age range 30–78 years; **Figure 1**); 329 and 150 patients underwent resection and MWA, respectively. Among 479 patients, 379(79.1%), 50 (10.4%), 10 (2.1%), and 40 (8.4%) patients had HBV-related, HCV-related, coinfection-related, and other HCC. There were 367 (76.6%) patients with cirrhosis and 222 (46.3%) patients with portal hypertension. In the TBS ≤3 cohort, there were 131, and 105 patients in the resection and MWA groups, respectively. In the TBS >3 cohort, there were 198, and 45 patients in the resection and MWA groups, respectively. After PSM, 83 patients each were in the resection and MWA groups in the TBS ≤3 cohort. In the TBS >3 cohort, there were 113, and 40 patients in the resection and MWA groups after PSM, respectively. After stabilized IPTW, in the TBS ≤3 cohort, there were 132.1 and 104.7 patients in the resection and MWA groups, respectively; in the TBS >3 cohort, there were 196.2 and 48.5 patients in the resection and MWA groups, respectively. No significant differences in sex; age; BMI; NLR; the presence of viral hepatitis; PLT count; AFP, ALB, TBIL, ALT, and AST levels; PT; Child–Pugh score; the presence cirrhosis; and the presence of hypersplenism were found whether in the total, PSM, or IPTW cohorts (**Tables 1**–**3**).

**Table 1:** Baseline characteristics by treatment group in total cohort.

Total cohort						
Variables	TBS ≤ 3 cohort				TBS > 3 cohort	
	RES group	MWA group	P value	RES group	MWA group	P value
	n = 131	n = 105		n = 198	n = 45	
**Age (years)**	55.97 (8.72)	56.4 (9.98)	0.724	58 (50,63)	59 (49,64)	0.367
Sex			0.508			0.645
Male	95 (72.5)	72 (68.6)		160 (80.8)	35 (77.8)	
Female	36 (27.5)	33 (31.4)		38 (19.2)	10 (22.2)	
BMI			0.774			0.006
≤24	81 (61.8)	63 (60.0)		115 (58.1)	16 (35.6)	
>24	50 (38.2)	42 (40.0)		83 (41.9)	29 (64.4)	
NLR			0.164			0.091
≤1.05	22 (16.8)	11 (10.5)		16 (8.1)	8 (17.8)	
>1.05	109 (83.2)	94 (89.5)		182 (91.9)	37 (82.2)	
AFP (ng/ml)			0.770			0.800
≤400	114 (87.0)	90 (85.7)		155 (78.3)	36 (80.0)	
>400	17 (13.0)	15 (14.3)		43 (21.7)	9 (20.0)	
Hepatopathy			0.650			0.894
No	7 (5.3)	3 (2.9)		26 (13.1)	4 (8.9)	
HBV	110 (84.0)	87 (82.9)		147 (74.2)	35 (77.8)	
HCV	12 (9.2)	12 (11.4)		21 (10.6)	5 (11.1)	
HBV+HCV	2 (1.5)	3 (2.9)		4 (2.0)	1 (2.2)	
Virus			0.519			0.435
No	7 (5.3)	3 (2.9)		26 (13.1)	4 (8.9)	
Yes	124 (94.7)	102 (97.1)		172 (86.9)	41 (91.1)	
ALB (g/L)			0.001			0.002
≤35	12 (9.2)	26 (24.8)		16 (8.1)	11 (24.4)	
>35	119 (90.8)	79 (75.2)		182 (91.9)	34 (75.6)	
**BILT (u mol/L)**	13 (10.1,17.4)	14.9 (10.1,22.9)	0.107	13.1 (9.5,17.4)	15.4 (10.3,20.6)	0.075
**ALT (U/L)**	30 (21,43)	31 (23,51)	0.415	32 (22,46)	38 (21,46)	0.075
**AST (U/L)**	37 (22,37)	31 (24,48.5)	**0.044**	28 (22,39)	33 (23,55)	**0.042**
**PT (s)**	12.2 (11.5,12.9)	12.6 (11.9,13.9)	**0.002**	12.0 (11.3,12.8)	12.7 (11.7,13.8)	**0.003**
**PLT (10^9)**	122 (89,157)	101 (60.5,139.5)	**0.001**	126.6 (93,173)	83 (58,141)	**0.001**
Child-Pugh			<0.001			0.003
A	123 (93.9)	77 (73.3)		186 (93.9)	36 (80.0)	
B	8 (6.1)	28 (26.7)		12 (6.1)	9 (20.0)	
Cirrhosis			0.045			0.046
No	31 (23.7)	14 (13.3)		60 (30.3)	7 (15.6)	
Yes	100 (76.3)	91 (86.7)		138 (69.7)	38 (84.4)	
Hypersplenism			<0.001			0.007
No	76 (58.0)	30 (28.6)		131 (66.2)	20 (44.4)	
Yes	55 (42.0)	75 (71.4)		67 (33.8)	25 (55.6)	

Bold values indicate P < 0.05.

**Table 2:** Baseline characteristics by treatment group in PSM cohort.

PSM cohort						
Variables	TBS ≤ 3 cohort			TBS > 3 cohort		
	RES group	MWA group	P value	RES group	MWA group	P value
	n = 83	n = 83		n = 113	n = 40	
**Age (years)**	55.13 (9.08)	56.27 (10.23)	0.452	59.00 (51.00,63.00)	57.50 (50.50,64.00)	0.988
Sex			0.868			0.506
Male	57 (68.7)	56 (67.5)		93 (82.3)	31 (77.5)	
Female	26 (31.3)	27 (32.5)		20 (17.7)	9 (22.5)	
BMI			0.524			0.132
≤24	53 (63.9)	49 (59.0)		58 (51.3)	15 (37.5)	
>24	30 (36.1)	34 (41.0)		55 (48.7)	25 (62.5)	
NLR			0.787			0.674
≤1.05	7 (8.4)	8 (9.6)		14 (12.4)	6 (15.0)	
>1.05	76 (91.6)	75 (90.4)		99 (87.6)	34 (85.0)	
AFP (ng/ml)			0.261			0.868
≤400	69 (83.1)	74 (89.2)		89 (78.8)	31 (77.5)	
>400	14 (16.9)	9 (10.8)		24 (21.2)	9 (22.5)	
Hepatopathy			0.663			0.255
No	2 (2.4)	3 (3.6)		17 (15.0)	3 (7.5)	
HBV	71 (85.5)	66 (79.5)		76 (67.3)	33 (82.5)	
HCV	9 (10.8)	11 (13.3)		16 (14.2)	4 (10.0)	
HBV+HCV	1 (1.2)	3 (3.6)		4 (3.5)	0 (0.0)	
Virus			1.000			0.224
No	2 (2.4)	3 (3.6)		17 (15.0)	3 (7.5)	
Yes	81 (97.6)	80 (96.4)		96 (85.0)	37 (92.5)	
ALB (g/L)			0.058			0.252
≤35	9 (10.8)	18 (21.7)		11 (9.7)	7 (17.5)	
>35	74 (89.2)	65 (78.3)		102 (90.3)	33 (82.5)	
**BILT (u mol/L)**	13.00 (10.55,17.20)	14.00 (9.80,18.40)	0.713	14.10 (11.10,18.80)	14.75 (10.17,20.00)	0.969
**ALT (U/L)**	30.00 (19.50,43.00)	30.00 (23.00,46.00)	0.493	32.00 (21.00,46.00)	37.00 (21.00,56.75)	0.339
**AST (U/L)**	26.00 (20.50,35.50)	30.00 (22.00,41.50)	0.149	29.00 (22.00,41.00)	31.50 (23.00,40.50)	0.631
**PT (s)**	12.20 (11.60,13.00)	12.40 (11.70,13.45)	0.266	12.30 (11.50,13.00)	12.60 (11.70,13.80)	0.129
**PLT (10^9)**	119.00 (85.00,154.00)	105.00 (65.00,150.50)	0.124	125.00 (84.00,167.00)	98.00 (67.50,153.25)	0.085
Child-Pugh			1.000			0.386
A	75 (90.4)	75 (90.4)		102 (90.3)	34 (85.0)	
B	8 (9.6)	8 (9.6)		11 (9.7)	6 (15.0)	
Cirrhosis			0.828			0.201
No	12 (14.5)	13 (15.7)		28 (24.8)	6 (15.0)	
Yes	71 (85.5)	70 (84.3)		85 (75.2)	34 (85.0)	
Hypersplenism			0.753			0.608
No	47 (56.6)	49 (59.0)		54 (47.8)	21 (52.5)	
Yes	36 (43.4)	34 (41.0)		59 (52.2)	19 (47.5)	

**Table 3:** Baseline characteristics by treatment group in Stabilized IPTW cohort.

Stabilized IPTW cohort						
Variables	TBS ≤ 3 cohort			TBS > 3 cohort		
	RES group	MWA group	P value	RES group	MWA group	P value
	n = 132.1	n = 104.7		n = 196.2	n = 48.5	
**Age (years)**	55.97 (8.69)	56.37 (10.50)	0.783	58.00 (50.00,63.00)	52.00 (49.00,62.66)	0.390
Sex			0.678			0.463
Male	95.6 (72.3)	73.2 (69.9)		158.1 (80.6)	41.3 (85.2)	
Female	36.5 (27.7)	31.5 (30.1)		38.1 (19.4)	7.2 (14.8)	
BMI			0.984			0.610
≤24	77.8 (58.9)	61.8 (59.0)		106.5 (54.3)	28.3 (58.4)	
>24	54.3 (41.1)	42.9 (41.0)		89.7 (45.7)	20.2 (41.6)	
NLR			0.830			1.000
≤1.05	17.0 (12.9)	12.5 (11.9)		18.9 (9.7)	4.9 (10.1)	
>1.05	115.1 (87.1)	92.2 (88.1)		177.3 (90.3)	43.6 (89.9)	
AFP (ng/ml)			0.876			0.757
≤400	114.6 (86.7)	90.1 (86.1)		154.2 (78.6)	39.1 (80.7)	
>400	17.5 (13.3)	14.6 (13.9)		42.0 (21.4)	9.4 (19.3)	
Hepatopathy			0.940			0.513
No	6.2 (4.7)	5.2 (4.9)		24.2 (12.3)	6.5 (13.4)	
HBV	108.5 (82.1)	85.5 (81.7)		146.6 (74.7)	39.2 (81.0)	
HCV	15.5 (11.7)	11.2 (10.7)		21.4 (10.9)	2.5 (5.2)	
HBV+HCV	1.9 (1.5)	2.8 (2.7)		4.0 (2.1)	0.2 (0.4)	
Virus			0.992			0.841
No	6.2 (4.7)	5.2 (4.9)		24.2 (12.3)	6.5 (13.4)	
Yes	125.9 (95.3)	99.5 (95.1)		172.0 (87.7)	42.0 (86.6)	
ALB (g/L)			0.255			0.119
≤35	19.0 (14.4)	20.9 (20.0)		18.6 (9.5)	8.4 (17.4)	
>35	113.1 (85.6)	83.8 (80.0)		177.6 (90.5)	40.1 (82.6)	
**BILT (u mol/L)**	14.10 (10.50,18.15)	13.60 (9.33,18.36)	0.615	13.20 (9.60,17.67)	13.95 (8.59,18.57)	0.685
**ALT (U/L)**	30.00 (21.00,43.00)	29.87 (22.50,47.30)	0.778	32.00 (22.48,46.00)	32.94 (20.80,45.68)	0.722
**AST (U/L)**	29.00 (22.00,39.67)	29.00 (21.45,41.88)	0.954	29.00 (22.00,39.17)	28.68 (20.52,34.10)	0.639
**PT (s)**	12.20 (11.60,13.30)	12.19 (11.70,13.40)	0.907	12.10 (11.30,12.90)	12.15 (11.60,12.81)	0.545
**PLT (10^9)**	109.61 (83.00,148.79)	113.15 (70.23,152.00)	0.708	125.00 (90.00,168.84)	93.42 (69.47,169.70)	0.308
Child-Pugh			0.851			1.000
A	110.6 (83.7)	88.6 (84.6)		179.8 (91.6)	44.5 (91.7)	
B	21.5 (16.3)	16.1 (15.4)		16.4 (8.4)	4.0 (8.3)	
Cirrhosis			0.863			0.707
No	26.8 (20.3)	22.2 (21.2)		54.2 (27.6)	12.1 (24.9)	
Yes	105.3 (79.7)	82.5 (78.8)		142.0 (72.4)	36.4 (75.1)	
Hypersplenism			0.844			0.410
No	72.1 (54.6)	55.8 (53.3)		84.8 (43.2)	17.8 (36.7)	
Yes	60.0 (45.4)	48.9 (46.7)		111.4 (56.8)	30.7 (63.3)	

### OS and PFS between resection and MWA in the TBS ≤3 cohort

**Figure 2:**
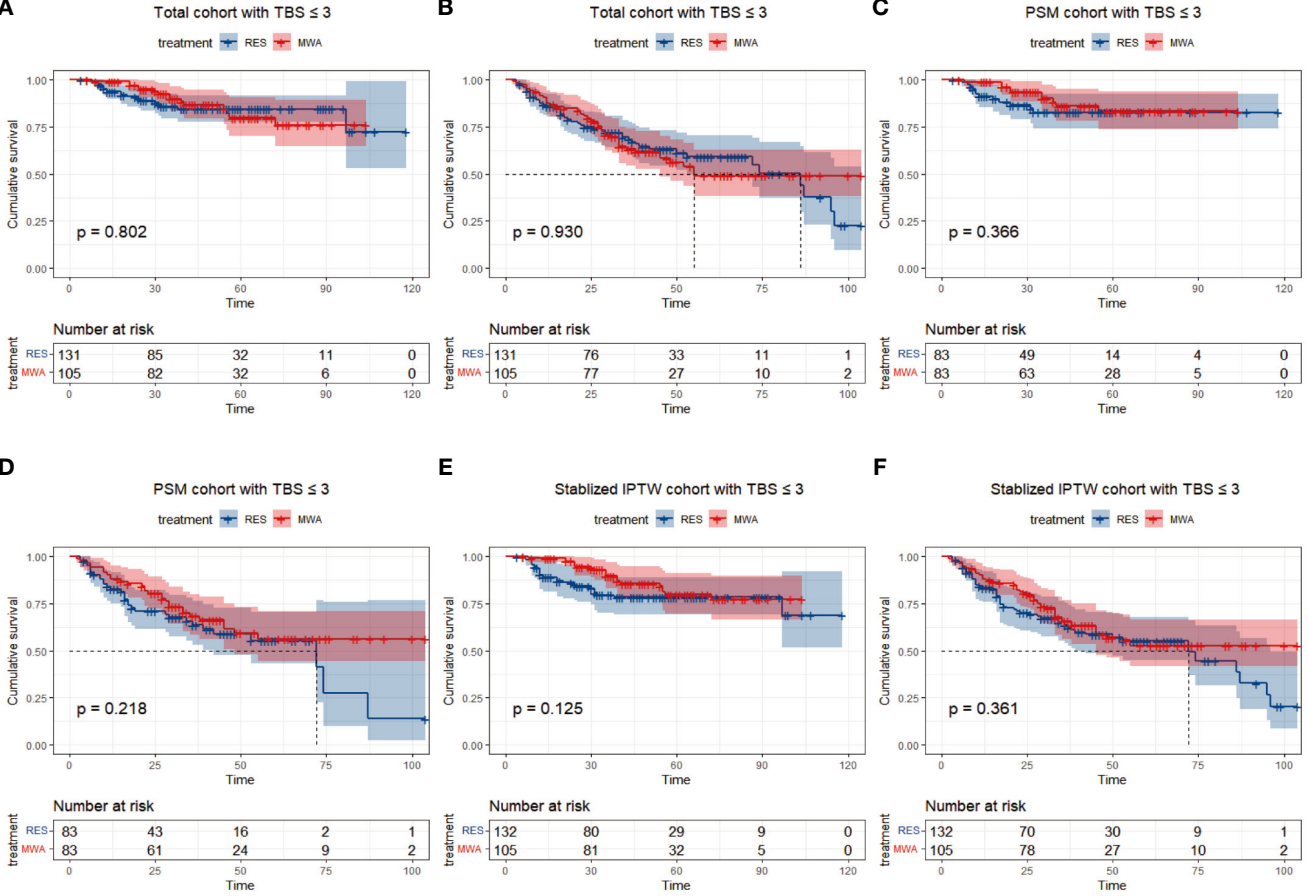
Differences of and overall survival **(A)** and progression-free survival **(B)** between RES group versus MWA group in total cohort with TBS ≤ 3. Differences of and overall survival **(C)** and progression-free survival **(D)** between RES group versus MWA group in PSM cohort with TBS ≤ 3. Differences of and overall survival **(E)** and progression-free survival **(F)** between RES group versus MWA group in Stabilized IPTW with TBS ≤ 3.

In the TBS ≤3 cohort, for the resection group, the median follow-up time was 46 months (range 4–118), 18/131 (13.7%) patients died, and 42/131 (36.6%) patients had tumor recurrence during the follow-up period. For the MWA group, the median follow-up time was 48 months (range 6–104), 15/105 (14.3%) patients died, and 42/105 (40%) patients had tumor recurrence during the follow-up period. Before PSM or stabilized IPTW, the 1-, 3-, and 5-year OS rates were 95.3%, 85.7%, and 84.4% in the resection group and 99.9%, 89.8%, and 79.7% in the MWA group, respectively (P = 0.802; **Figure 2A**). The 1-, 3-, and 5-year PFS rates were 86%, 68.3%, and 59.2% in the resection group and 87.6%, 63.3%, and 49.0% in the MWA group, respectively (P = 0.930; **Figure 2B**). Following PSM, the 1-, 3-, and 5-year OS rates were 92.5%, 82.7%, and 82.7% in the resection group and 98.8%, 90.0%, and 83.2% in the MWA group, respectively (P = 0.366; **Figure 2C**); the corresponding PFS rates were 82.7%, 63.6%, and 55.2% and 88.0%, 68.3%, and 56.3% in the resection and MWA groups, respectively (P = 0.218; **Figure 2D**). Following stabilized IPTW, the 1-, 3-, and 5-year OS rates were 90.3%, 79.5%, and 78.4% in the resection group and 98.9%, 89.5%, and 79.7% in the MWA group, respectively (P = 0.125; **Figure 2E**); the corresponding PFS rates were 83.4%, 63.5%, and 55.0% and 87.9%, 65.5%, and 52.7% in the resection and MWA groups, respectively (P = 0.361; **Figure 2F**). In conclusion, there were no significant differences in OS and PFS between the resection and MWA groups in the TBS ≤3 cohort.

### OS and PFS between resection and MWA in the TBS >3 cohort

**Figure 3:**
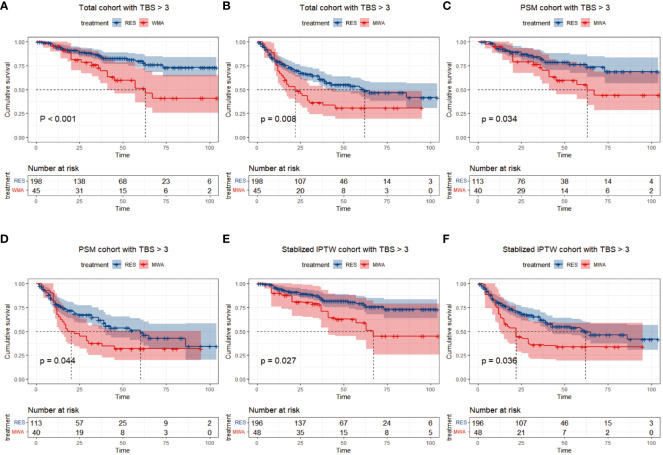
Differences of and overall survival **(A)** and progression-free survival **(B)** between RES group versus MWA group in total cohort with TBS > 3. Differences of and overall survival **(C)** and progression-free survival **(D)** between RES group versus MWA group in PSM cohort with TBS >3. Differences of and overall survival **(E)** and progression-free survival **(F)** between RES group versus MWA group in Stabilized IPTW cohort with TBS > 3.

In the TBS >3 cohort, for the resection group, the median follow-up time was 44 months (range 1–104), 32/198 (16.2%) patients died, and 80/198 (40.4%) patients had tumor recurrence during the follow-up period. For the MWA group, the median follow-up time was 62 months (range 4–105), 19/45 patients (42.2%) died, and 30/45 (66.7%) patients had tumor recurrence during the follow-up period. Before PSM or stabilized IPTW, the 1-, 3-, and 5-year OS rates were 94.0%, 86.0%, and 79.7% in the resection group and 95.6%, 72.7%, and 51.3% in the MWA group, respectively (P < 0.001; **Figure 3A**). The 1-, 3-, and 5-year PFS rates were 79.4%, 61.2%, and 50.5% in the resection group and 68.9%, 36.3%, and 30.8% in the MWA group, respectively (P = 0.008; **Figure 3B**). Following PSM, the 1-, 3-, and 5-year OS rates were 92.5%, 82.8%, and 76.3% in the resection group and 95.0%, 73.2%, and 55.1% in the MWA group, respectively (P = 0.034; **Figure 3C**); the corresponding PFS rates were 78.0%, 61.6%, and 48.6% and 67.5%, 37.5%, and 31.7% in the resection and MWA groups, respectively (P = 0.044; **Figure 3D**). Following stabilized IPTW, the 1-, 3-, and 5-year OS rates were 94.1%, 85.9%, and 79.1% in the resection group and 89.8%, 77.7%, and 58.9% in the MWA group, respectively (P = 0.027; **Figure 3E**); the corresponding PFS rates were 79.8%, 62.4%, and 50.9% and 68.0%, 35.8%, and 33.9% in the resection and MWA groups, respectively (P = 0.036; **Figure 3F**). In conclusion, there were significant differences in OS and PFS between the resection and MWA groups in the TBS >3 cohort.

### Sensitivity analysis

Multivariate analysis showed that ALB >35 g/L (hazard ratio [HR] 95CI% 0.25 [0.12–0.52], P < 0.001) and cirrhosis (HR 95CI% 2.69 [1.23–5.91], P = 0.013) were associated with a better OS, and male sex (HR 95CI% 1.65 [1.01–2.69], P = 0.047) and NLR >1.05 (HR 95CI% 3.09 [1.34–7.09], P = 0.008) were associated with a poorer PFS in the TBS ≤3 cohort (**Supplementary Table 1**). Additionally, in the TBS >3 cohort, age ≥60 years (HR 95CI% 2.12 [1.21–3.71], P = 0.008), TBIL >17.1 µmol/L (HR 95CI% 1.78 [1.01–3.15], P = 0.048), and PLT <100 ∗ 10^9^ (HR 95CI% 2.87 [1.61–5.3], P < 0.001) were associated with a poorer OS, and viral hepatitis (HR 95CI% 2.59 [1.19–5.60], P = 0.016) and TBIL >17.1 µmol/L (HR 95CI% 1.68 [1.13–2.51], P = 0.011) were associated with a poorer PFS (**Supplementary Table 1**).

In the TBS ≤3 cohort, the type of surgical modality was not associated with poorer OS or PFS, no matter how many factors were adjusted (**Supplementary Figure 1A**). In contrast, in the TBS >3 cohort, the type of surgical modality was associated with poorer OS or PFS, no matter how many factors were adjusted (**Supplementary Figure 1B**).

## Discussion

HCC is a highly heterogeneous disease in terms of biological and clinical behavior (19). Systemic therapy has been shown to prolong the survival of patients with advanced stage HCC (20, 21). The 2022 version of BCLC staging demonstrates that both ablation and liver resection are clinically effective treatment options and have comparable therapeutic effects for very early and early-stage HCC (2). MWA has become an increasingly used local ablation modality. Its theoretical benefits include high thermal efficiency and a larger ablation zone compared with radiofrequency ablation (22). To date, the best approach to the management of HCC within the Milan criteria, eligible for both microwave coagulation and liver resection, remains controversial.

According to previous studies, tumor morphology has been validated as a strong predictor of recurrence and poorer survival outcomes (23, 24). Sun et al. reported that no marked difference was found in the 1-, 3-, and 5-year OS rates and the 1-year disease-free survival (DFS) rate between the MWA and resection groups. Additionally, no significant differences were observed in the OS and DFS rates between the two groups with solitary HCC ≤3 cm and in the OS rate for solitary HCC 3–5 cm (25). Nevertheless, the DFS for solitary HCC 3–5 cm in the resection group was significantly higher compared with that in the MWA group in the study cited above. Interestingly, a study demonstrated that in the subgroup of BCLC-0, no significant differences in PFS or OS were observed between the MWA and liver resection groups. Conversely, in the subgroup of BCLC-A, the liver resection group had a significant increase in PFS compared with the MWA group (26). Of note, a cohort study by Dou et al. demonstrated that no differences were observed regarding OS and DFS in HCC ≤4.0 cm after MWA or surgical resection. For HCC 4.1–5.0 cm, MWA had lower OS (P = 0.01) and DFS rates (P = 0.01) than surgical resection (27). These results indicate that tumor burden might be a reliable tool to differentiate the prognosis in patients with HCC after MWA and resection.

Tumor burden is considered one of the most important prognostic predictors of HCC (14, 17, 28). Traditionally, the tumor diameter, and number of nodules are used to assess tumor burden. Although the use of arbitrary cutoff categorical (tumor size) or ordinal (tumor number) values is a convenient way to assess disease burden, it has limitations with regard to statistical power compared with continuous variables (12). Previous studies have suggested the use of total tumor volume and diameter, which are continuous variables, to assess tumor burden (29, 30). However, these two scores are too complicated because of the requirement of all tumor number and size information. The use of the Pythagorean theorem, with TBS as a single and continuous variable rather than a dichotomous variable to indicate disease burden in HCC, has recently been proposed to minimize the heterogeneity in tumor nodule size and number. Previous studies demonstrate that TBS has a better predictive ability for outcomes compared with the established Milan or up-to-7 criteria (12, 17). Additionally, some studies suggest TBS as a discriminator for some treatment decisions in HCC (18, 31).

This study noted that TBS is a feasible marker to discriminate long-term outcomes, and we noted that TBS may provide a differential influence in selecting resection or MWA for HCC within the Milan criteria. In the TBS ≤3 cohort, there was no significant difference in PFS and OS between the two groups. However, in the TBS >3 cohort, PFS, and OS rates were higher in the resection group than in the MWA group. After PSM or stabilized IPTW, similar results were observed in the TBS ≤3 and TBS >3 cohorts. Additionally, after multivariate Cox regression model adjustments, surgical modalities were not associated with a poorer prognosis in the low TBS cohort but were associated with a poorer prognosis in the high TBS cohort.

Our study findings have some limitations. First, this was a retrospective single-center clinical study. Although the IPTW and PSM analyses were conducted to reduce selection and confounding biases, potential flaws may still exist. Second, the patient number in the MWA group was small for the TBS >3 cohort. Third, we have no reliable data on the treatment of patients with recurrent disease and it is a possible additional confounder in the analysis of long-term survival and PFS. In addition, TBS = 3 was not the best cutoff value of prognostic outcomes in our study. Hence, future multi-center prospective studies are needed to be validated.

In conclusion, preoperative TBS as a discriminator might help guide treatment decision-making for HCC within the Milan criteria and in the group with TBS >3, surgery can be considered in patients requiring a less extensive surgery.

## Data availability statement

The raw data supporting the conclusions of this article will be made available by the authors, without undue reservation.

## Ethics statement

The studies involving humans were approved by the ethics committee of Shengjing Hospital of China Medical University (2023PS760K). The studies were conducted in accordance with the local legislation and institutional requirements. The participants provided their written informed consent to participate in this study. Written informed consent was obtained from the individual(s) for the publication of any potentially identifiable images or data included in this article.

## Author contributions

CD: Funding acquisition, Resources, Supervision, Writing – review & editing. ZW: Conceptualization, Data curation, Investigation, Methodology, Writing – original draft. KX: Conceptualization, Data curation, Investigation, Methodology, Writing – original draft. FX: Supervision, Writing – original draft.
