# Supplementary material for: The tumor burden score may be a discriminator in microwave ablation versus liver resection for hepatocellular carcinoma within the Milan criteria: a propensity score matching and inverse probability of treatment weighting study
Source: Front Oncol. 2024 Feb 16;14:1330851. doi: 10.3389/fonc.2024.1330851 (PMC10905383; doi:10.3389/fonc.2024.1330851)

**Supplementary Table 1.** Cox regression

| Total cohort with TBS ≤ 3 | | | | | | | | | | |
| --- | --- | --- | --- | --- | --- | --- | --- | --- | --- | --- |
|  | Overall survival |  |  |  |  |  | Progression-free survival | | | |
|  | Univariate |  |  | Multivariate |  |  | Univariate |  | Multivariate |  |
|  | HR (95CI%) | P |  | HR (95CI%) | P |  | HR (95CI%) | P | HR (95CI%) | P |
| Age (≥60 years) | 1.46(0.74–2.89) | 0.280 |  |  |  |  | 0.72(0.47–1.11) | 0.138 |  |  |
| Sex (Male) | 1.25(0.57–2.78) | 0.579 |  |  |  |  | 1.52(0.93–2.48) | 0.093 | 1.65(1.01–2.69) | **0.047** |
| BMI (>24 Kg/m^2^) | 1.01(0.50–2.02) | 0.988 |  |  |  |  | 1.00(0.65–1.53) | 0.997 |  |  |
| NLR (>1.05) | 2.62(0.63–10.95) | 0.187 |  |  |  |  | 2.87(1.25–6.59) | **0.013** | 3.09(1.34–7.09) | **0.008** |
| AFP (>400 ng/ml) | 1.83(0.79–4.22) | 0.157 |  |  |  |  | 1.47(0.84–2.56) | 0.179 |  |  |
| Virus (Yes) | 0.45(0.11–1.87) | 0.270 |  |  |  |  | 3.09(0.43–22.16) | 0.263 |  |  |
| ALB (>35 g/L) | 0.31(0.15–0.63) | **0.001** |  | 0.25(0.12–0.52) | **<0.001** |  | 0.67(0.40–1.13) | 0.131 |  |  |
| BILT (>17.1u mol/L) | 2.03(1.02–4.02) | **0.043** |  |  |  |  | 1.12(0.72–1.74) | 0.611 |  |  |
| ALT (> 40 U/L) | 1.47(0.74–2.93) | 0.277 |  |  |  |  | 1.01(0.65–1.56) | 0.983 |  |  |
| AST (>40 U/L) | 1.38(0.67–2.85) | 0.380 |  |  |  |  | 1.17(0.74–1.86) | 0.509 |  |  |
| PLT (< 100*10^9) | 0.77(0.39–1.54) | 0.464 |  |  |  |  | 1.22(0.80–1.55) | 0.360 |  |  |
| Child-Pugh (B grade) | 2.27(1.05–4.88) | **0.037** |  |  |  |  | 1.52(0.90–2.54) | 0.117 |  |  |
| Cirrhosis (Yes) | 0.52(0.25–1.09) | 0.081 |  | 0.37(0.17–0.82) | **0.013** |  | 1.09(0.63–1.87) | 0.766 |  |  |
| Hypersplenism (Yes) | 0.57(0.28–1.17) | 0.126 |  |  |  |  | 0.80(0.53–1.22) | 0.309 |  |  |
| Total cohort with TBS > 3 | | | | | | | | | | |
| Age (≥60 years) | 2.07(1.19–3.59) | **0.010** |  | 2.12(1.21–3.71) | **0.008** |  | 1.04(0.71–1.52) | 0.831 |  |  |
| Sex (Male) | 1.07(0.52–2.19) | 0.864 |  |  |  |  | 0.89(0.54–1.45) | 0.628 |  |  |
| BMI (>24 Kg/m^2^) | 0.73(0.42–1.30) | 0.285 |  |  |  |  | 0.97(0.67–1.42) | 0.883 |  |  |
| NLR (>1.05) | 2.05(0.64–6.59) | 0.229 |  |  |  |  | 1.46(0.71–3.01) | 0.300 |  |  |
| AFP (>400 ng/ml) | 1.58(0.86–2.89) | 0.138 |  |  |  |  | 1.52(0.99–2.33) | 0.058 |  |  |
| Virus (Yes) | 1.15(0.45–2.89) | 0.775 |  |  |  |  | 2.27(1.05–4.87) | **0.036** | 2.59(1.19–5.60) | **0.016** |
| ALB (>35 g/L) | 0.86(0.39–1.91) | 0.710 |  |  |  |  | 1.03(0.57–1.88) | 0.913 |  |  |
| BILT (>17.1u mol/L) | 2.36(1.35–4.10) | **0.002** |  | 1.78(1.01–3.15) | **0.048** |  | 1.52(1.02–2.26) | **0.038** | 1.68(1.13–2.51) | **0.011** |
| ALT (> 40 U/L) | 1.12(0.64–1.96) | 0.699 |  |  |  |  | 1.22(0.83–1.79) | 0.309 |  |  |
| AST (>40 U/L) | 1.91(1.08–3.40) | **0.027** |  |  |  |  | 1.18(0.76–1.82) | 0.458 |  |  |
| PLT (< 100*10^9) | 3.11(1.78–5.45) | **<0.001** |  | 2.87(1.61–5.3) | **<0.001** |  | 1.36(0.93–2.00) | 0.115 |  |  |
| Child-Pugh (B grade) | 2.64(1.32–5.28) | **0.006** |  |  |  |  | 1.46(0.82–2.60) | 0.204 |  |  |
| Cirrhosis (Yes) | 3.35(1.33–8.43) | **0.010** |  |  |  |  | 1.36(0.86–2.16) | 0.186 |  |  |
| Hypersplenism (Yes) | 0.53(0.30–0.92) | **0.024** |  |  |  |  | 0.82(0.57–1.20) | 0.314 |  |  |
| Abbreviation: concentrations of α-fetoprotein: AFP; albumin: ALB; alanine aminotransferase: ALT; aspartate aminotransferase: AST; body mass index: BMI; microwave ablation: MWA; neutrophil to lymphocyte ratio: NLR; platelet count: PLT; resection: RES; prothrombin time: PT; total bilirubin: TBIL; Tumor burden: TBS. | | | | | | | | | | |

**Supplementary Figure 1**


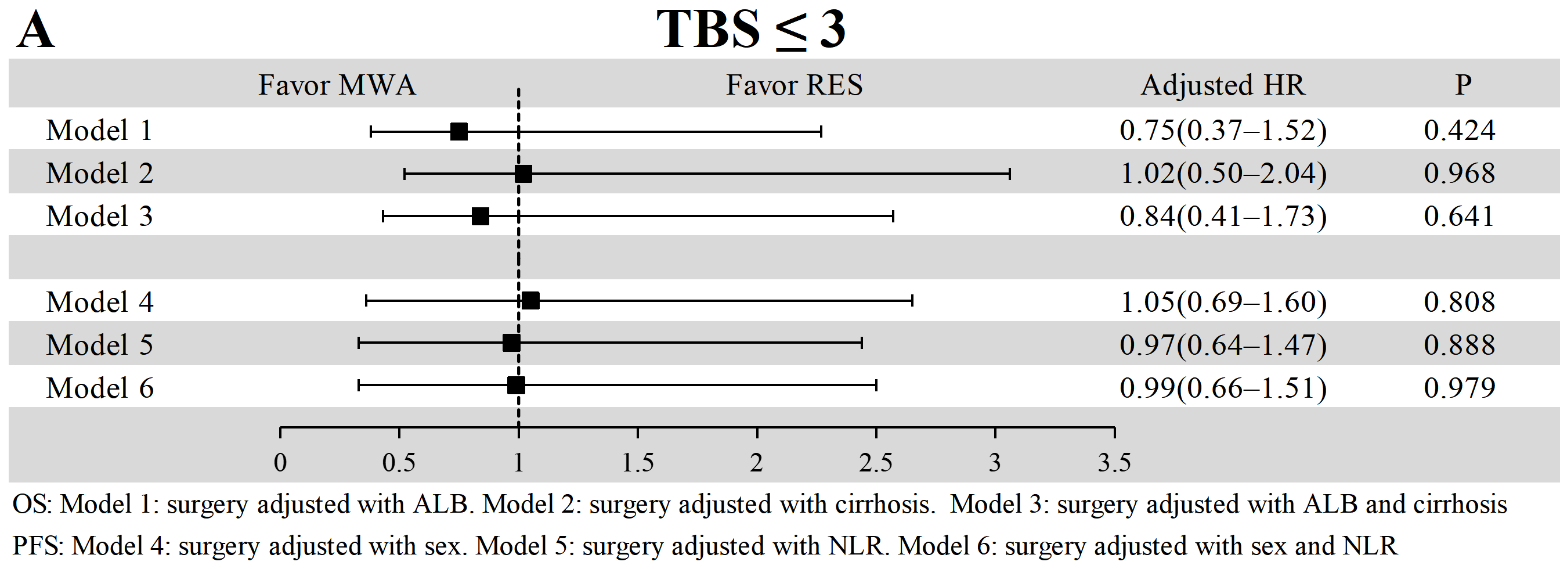


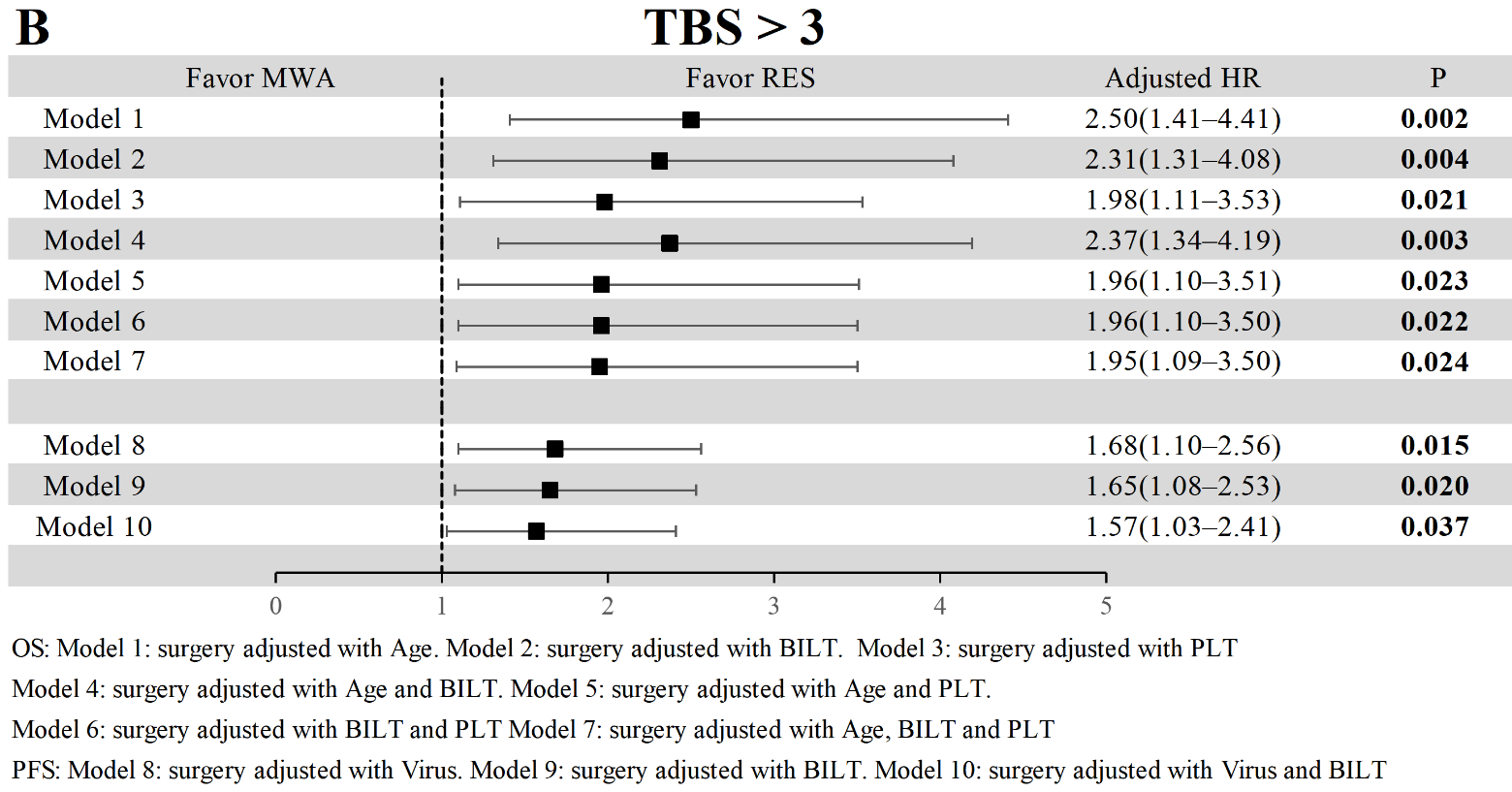

Supplement: Supplementary file 1 [file DataSheet_1.docx]
